# Multisource surveillance conducted by the Tokyo Metropolitan Government during the Tokyo 2020 Olympic and Paralympic Games

**DOI:** 10.5365/wpsar.2023.14.3.978

**Published:** 2023-09-30

**Authors:** Yoshiyuki Sugishita, Yoshiko Somura, Nobuyuki Abe, Yasuko Murai, Yoshiaki Koike, Eriko Suzuki, Mayu Yanagibayashi, Aya Kayebeta, Atsushi Yoshida

**Affiliations:** a Infectious Disease Control Division, Bureau of Social Welfare and Public Health, Tokyo Metropolitan Government, Tokyo, Japan.; b Epidemiological Information Section, Tokyo Metropolitan Institute of Public Health, Tokyo, Japan.

## Abstract

The Tokyo 2020 Olympic and Paralympic Games (the Games) were held from 23 July to 5 September 2021 in Tokyo, Japan, after a 1-year delay due to the coronavirus disease (COVID-19) pandemic. The Tokyo Metropolitan Government was responsible for monitoring and responding to infectious disease outbreaks other than COVID-19 during the Games. A multisource surveillance system was used from 1 July to 12 September 2021 for the early detection and rapid response to infectious diseases. This included routine notifiable disease surveillance, sentinel surveillance, syndromic surveillance, cluster surveillance, ambulance transfer surveillance and the Tokyo Infectious Alert system. Daily reports were disseminated summarizing the data collected from the multisource surveillance system. No case of infectious disease under the Tokyo Metropolitan Government system required a response during the Games. The multisource surveillance was useful for providing intelligence during the Games and, if required, could contribute to the early detection and rapid response to outbreaks during other mass gatherings. The system could be improved to overcome the challenges implied by the findings of this multisource surveillance.

Outbreaks of infectious disease, widespread food poisoning incidents and bioterrorism attacks are more probable during mass-gathering events such as the Olympic and Paralympic Games. (*1*) The rapid detection of infectious diseases, potential outbreaks and other public health events at mass gatherings is crucial. Surveillance during mass gatherings is typically conducted by public health agencies that adopt additional surveillance mechanisms to augment their routine public health surveillance during the event.

The Tokyo 2020 Olympic and Paralympic Games (the Games) were held from 23 July to 5 September 2021, the 1-year delay being due to the novel coronavirus disease (COVID-19) pandemic. The Games were held during the fifth wave of COVID-19 in Japan with the arrival of the Delta variant strain while the public health response to COVID-19 was ongoing. Consequently, only players and staff members were in attendance. The Tokyo Organizing Committee of the Olympic and Paralympic Games was responsible for monitoring and responding to COVID-19 during the Games, and the Tokyo Metropolitan Government (TMG) was responsible for monitoring and responding to all infectious diseases other than COVID-19.

TMG has conducted similar surveillance for mass gatherings including during the 2013 Sports Festival in Tokyo (*2*) and the 2019 Rugby World Cup in Japan. (*3*) Lessons learned from these earlier mass-gathering events were used to develop the mechanisms for the Games. This report summarizes the multisource surveillance conducted by TMG for infectious diseases other than COVID-19 during the Games.

## Methods

### Surveillance systems

Multisource surveillance for infectious diseases other than COVID-19 for the Games was conducted by TMG from 1 July to 12 September 2021, including national holidays and weekends. Data were sourced from the following surveillance systems.

#### Official notifiable disease surveillance

In compliance with the Act on the Prevention of Infectious Diseases and Medical Care for Patients with Infectious Diseases (the Infectious Diseases Control Law), notifiable disease surveillance requires diagnosing physicians to notify authorities of 87 diseases across five severity categories. For the Games, official notifiable disease surveillance included all diseases in categories I–IV with the exclusion of tuberculosis and three diseases in Category V (invasive meningococcal infection, measles, rubella) (**Table 1**). Notifications from the diagnosing physicians are usually published by TMG once a week; however, during the Games, they were reported in the daily Games report.

**Table 1:** Infectious diseases in the notifiable diseases surveillance system by severity category included in surveillance for the Tokyo 2020 Olympic and Paralympic Games, 1 July–12 September 2021

Category	Diseases
I	Crimean-Congo haemorrhagic fever, Ebola haemorrhagic fever, Lassa fever, Marburg disease, plague, smallpox, South American haemorrhagic fever
II	Acute poliomyelitis, avian influenza (H5N1), avian influenza (H7N9), diphtheria, Middle East respiratory syndrome (only if the pathogen is MERS coronavirus), severe acute respiratory syndrome (only if the pathogen is SARS coronavirus)
III	Cholera, Enterohaemorrhagic *Escherichia coli* infection, paratyphoid fever, shigellosis, typhoid fever
IV	Anthrax, avian influenza (excluding H5N1 or H7N9), B virus disease, botulism, brucellosis, Chikungunya fever, coccidioidomycosis, dengue fever, Eastern equine encephalitis, echinococcosis, epidemic typhus, glanders, haemorrhagic fever with renal syndrome, Hantavirus pulmonary syndrome, Hendra virus infection, hepatitis A, hepatitis E, Japanese encephalitis, Japanese spotted fever, Kyasanur Forest disease, legionellosis, leptospirosis, Lyme disease, lyssavirus infection, malaria, melioidosis, monkeypox, Nipah virus infection, Omsk haemorrhagic fever, psittacosis, Q fever, rabies, relapsing fever, Rift Valley fever, Rocky Mountain spotted fever, severe fever with thrombocytopenia syndrome (only if the pathogen is SFTS virus of the genus *Phlebovirus*), tick-born encephalitis, Tsutsugamushi disease, tularaemia, Venezuelan equine encephalitis, West Nile fever, Western equine encephalitis, yellow fever, Zika virus infection
V	Invasive meningococcal infection, measles, rubella

#### Official sentinel surveillance

In compliance with the Infectious Diseases Control Law, the sentinel surveillance system receives weekly reports from different types of sentinel sites. These reports comprise daily data collected Monday to Sunday by each site on 1–12 diseases, submitted on a weekly basis to their local public health centre (**Table 2**). During the Games, sentinel data continued to be reported weekly and were included only in the daily Games reports issued on Thursdays.

**Table 2:** Types of sentinel sites and the diseases included in their sentinel surveillance for the Tokyo 2020 Olympic and Paralympic Games, 1 July–12 September 2021

Classification of sentinel sites (subject)	No. of sites	Diseases
Paediatric sentinel sites (paediatric medical facilities)	264	Erythema infectiosum, exanthema subitum, group A streptococcal pharyngitis, hand, foot and mouth disease, herpangina, infectious gastroenteritis, Kawasaki disease,^a^ mumps, pharyngoconjunctival fever, respiratory syncytial virus infection, undiagnosed exanthems,^a^ varicella
Influenza sentinel sites (internal and paediatric medical facilities)	419	Influenza (excluding avian influenza and pandemic influenza [novel influenza or re-emerging influenza])
Ophthalmology sentinel sites (ophthalmologic facilities)	39	Acute haemorrhagic conjunctivitis, epidemic keratoconjunctivitis
General hospital sentinel sites (medical facilities, each with at least 300 beds)	25	Aseptic meningitis, bacterial meningitis (excluding cases for which the cause is identified as *Haemophilus influenzae*, *Neisseria meningitides* or *Streptococcus pneumoniae*), chlamydial pneumonia (excluding psittacosis), infectious gastroenteritis (only if the pathogen is rotavirus), influenza (excluding avian influenza and pandemic influenza [novel influenza or re-emerging influenza]), mycoplasma pneumonia

^a^ Kawasaki disease and undiagnosed exanthems are diseases designated by the Tokyo Metropolitan Government; they are not based on the Act on the Prevention of Infectious Diseases and Medical Care for Patients with Infectious Diseases.

#### Official syndromic surveillance

In accordance with the Infectious Diseases Control Law, syndromic surveillance of cases of unknown pathogens is used to detect outbreaks. The case definitions include: a patient with fever, respiratory symptoms, rash, gastrointestinal symptoms, neurological symptoms, or other symptoms suggestive of some infectious disease; a patient needing intensive care; and a physician unable to diagnose the disease immediately based on generally accepted medical knowledge. During the Games, 36 hospitals from this system, plus two new temporary hospitals, were designated as reporting sites for syndromic surveillance.

#### Cluster surveillance

Cluster surveillance is conducted at residential aged-care facilities and nursery schools based on the requirements of the central government. From January 2019, TMG extended its cluster surveillance and also mandated schools, medical institutions and any other facility to notify their local public health centre when more than 10 people report or are diagnosed with the same symptom or when more than half of the users and staff of a facility share the same symptom or diagnosis during the previous 7 days. Initially, for the Games, public health centres were asked to report their cluster surveillance weekly as usual; however, this was revised to daily reporting from 11 July 2021 onwards.

#### Ambulance transfer surveillance

The ambulance transfer surveillance system is unique to TMG. It collects and analyses information at the time of emergency transport using data provided by the Tokyo Fire Department’s Emergency Information Analysis and Management System. (*4*) It operated as usual during the Games.

#### The Tokyo Infectious Alert system

The Tokyo Infectious Alert system, another unique system operated by TMG, has been used for avian influenza (H5N1 and H7N9), Middle East respiratory syndrome (MERS) and severe acute respiratory syndrome (SARS). Doctors notify suspected cases of severe respiratory syndrome to public health centres, which collect and transfer the specimens for testing. The test results of suspected cases are delivered 24 hours a day to public health centres and medical facilities through the infectious disease control unit of TMG. This system operated as usual during the Games.

#### Prescription and absenteeism surveillance systems

The prescription surveillance system is a nationwide syndromic surveillance system established in 2009 that reports on the estimated number of influenza and chickenpox cases and the number of patients prescribed certain types of drugs, based on pharmacy prescriptions. (*5*, *6*) In 2021, approximately 12 000 pharmacies participated, collectively accounting for approximately 20% of all pharmacies nationwide. The estimated numbers of patients are published online each morning.

Information related to school or nursery school absenteeism is integrated and systematized into the Nursery School Absenteeism Surveillance System ((N)SASSy). (*7*-*9*) In 2021, approximately half (22 000) of all schools, including kindergartens, as well as approximately one fourth (7100) of nursery schools in Japan, reported to the system, thus monitoring the daily health of approximately 6 million children 18 years old or younger nationwide.

During the Games, TMG monitored prescription surveillance and (N)SASSy more closely than usual.

### Procedures

**Fig. 1:**
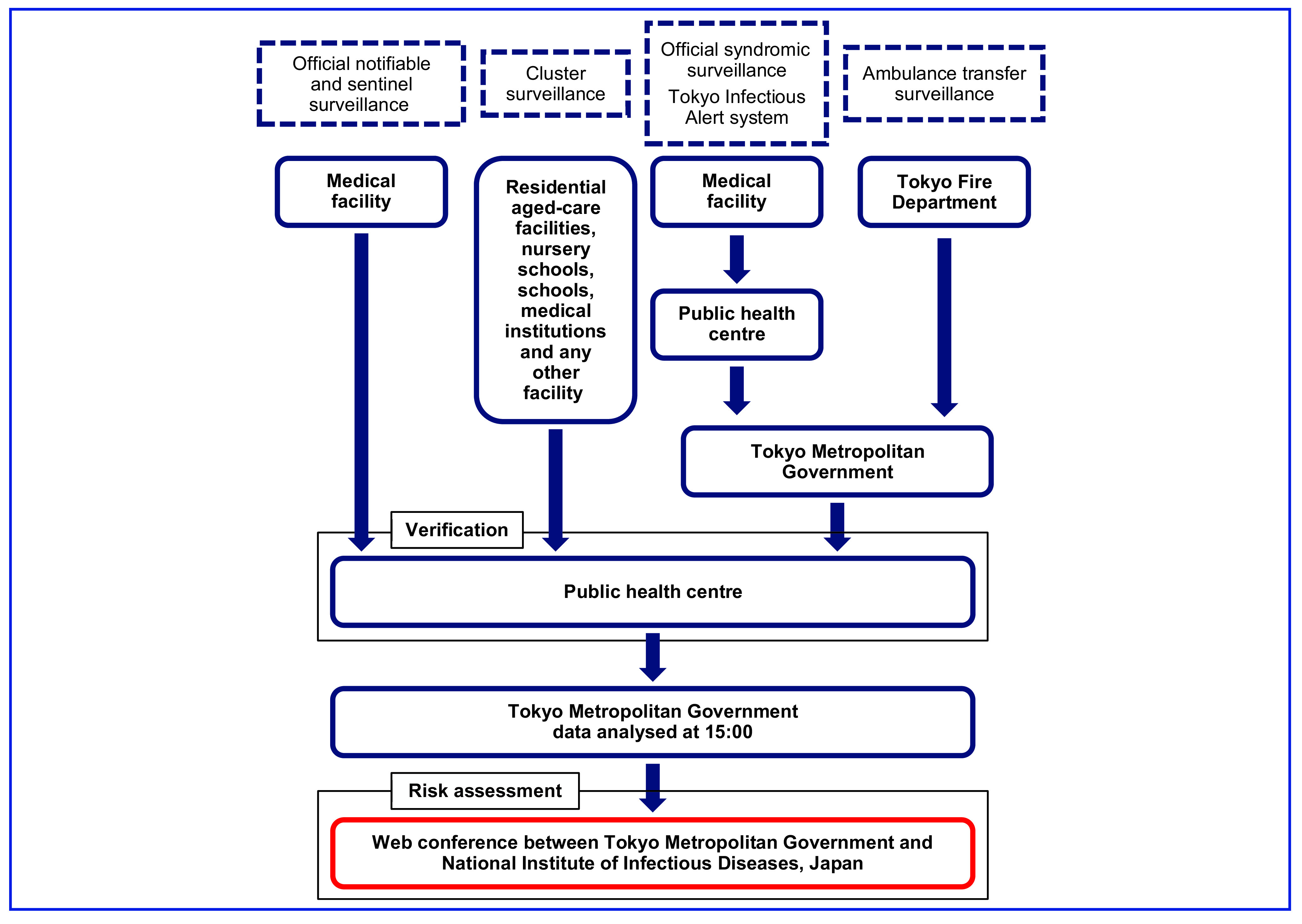
Flowchart of surveillance for the Tokyo 2020 Olympic and Paralympic Games by the Tokyo Metropolitan Government, 1 July–12 September 2021

Jurisdictional public health centres verified outbreak information obtained from official notifiable surveillance, official sentinel surveillance and cluster surveillance (**Fig. 1**). Data from the official syndromic surveillance and Tokyo Infectious Alert system were reported to TMG by public health centres, as were ambulance transfer surveillance data from the Tokyo Fire Department’s system. The public health centres were involved in the verification of these surveillance systems.

[**insert** Figure 1]

Risk assessment was performed by TMG with the National Institute of Infectious Diseases, Japan (NIID) following WHO guidelines for risk assessment. (*10*) In particular, the importance for public health was assessed in terms of severity and/or infectivity while the possibility of outbreak expansion was assessed based on the location, environment, timing and extent of occurrence.

Data from all surveillance systems were collated by TMG, with 15:00 as the daily cut-off time for reporting (**Fig. 1**). The data were summarized into daily reports which were assessed and confirmed at daily web conferences with NIID. The final report was delivered to all relevant stakeholders, including the public health centre in Tokyo, the Tokyo Medical Association, the Ministry of Health, Labour and Welfare, and the Tokyo Organizing Committee of the Olympic and Paralympic Games, with 17:00 as the daily cut-off time. The daily report was not provided to the general public.

### Response plan

**Fig. 2:**
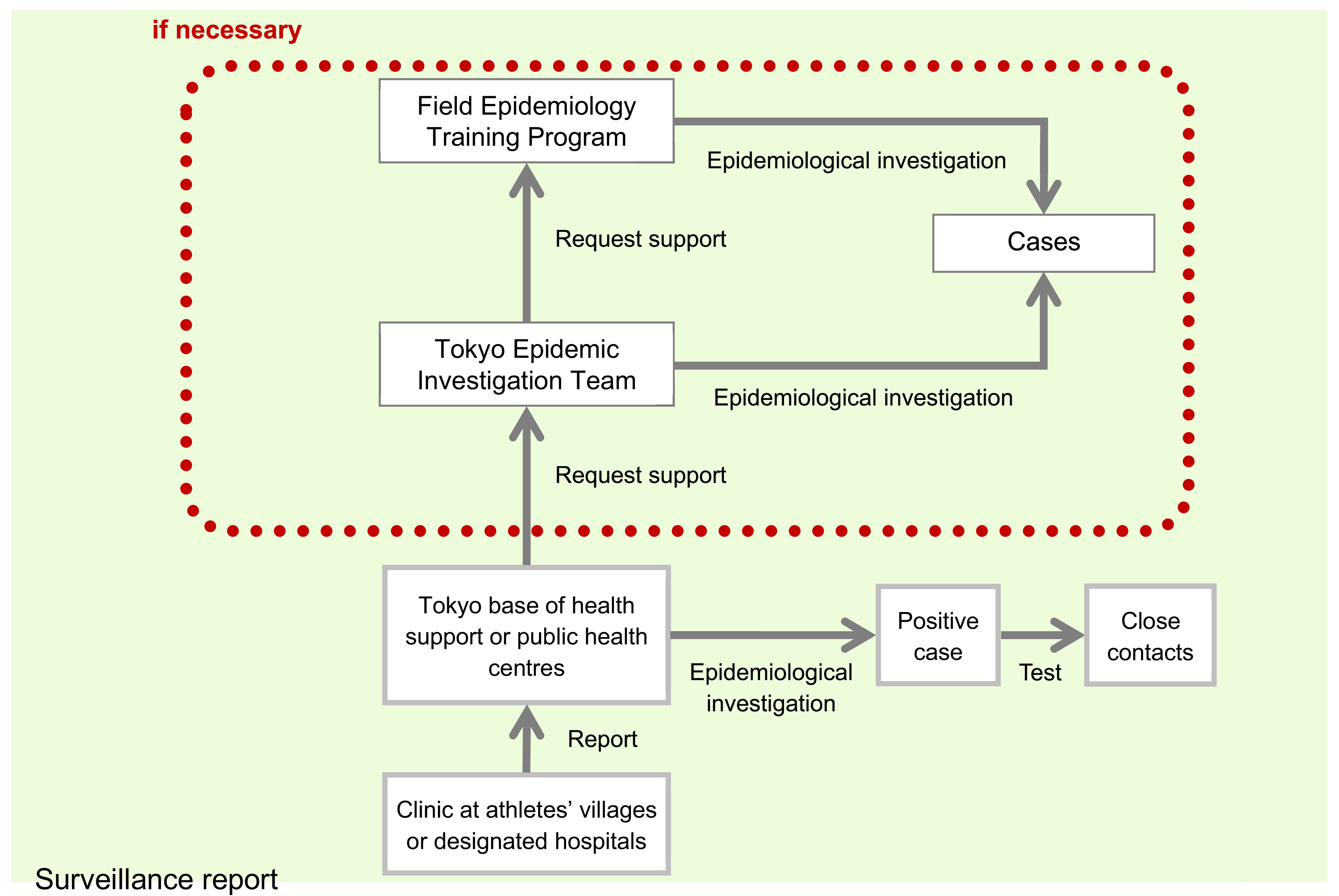
Response plan for potential investigations during the Tokyo 2020 Olympic and Paralympic Games, 1 July–12 September 2021

Outbreak investigations within the athletes’ village were the responsibility of the Tokyo Base of Health Support, which was responsible for providing public health services to the athletes’ village during the Games. It could request support from the Tokyo Epidemic Investigation Team and the Field Epidemiology Training Program at NIID, if required (**Fig. 2**). Evaluations of unusual events and the determination of appropriate responses were conducted at the daily web conferences between TMG and NIID.

[**insert** Figure 2]

## Results

### Preparation

Preparations for surveillance of the Games commenced in April 2021. Weekly meetings were held between the relevant TMG units and organizations including the Tokyo Organizing Committee of the Olympic and Paralympic Games and the Ministry of Health, Labour and Welfare from 25 May 2021. Two practice sessions for producing daily reports were held from 19 to 20 May and from 18 to 24 June 2021. The multisource surveillance system was set up in 3 months, including system user guides.

### Surveillance system operations

The surveillance report was published daily during the Games as planned. Mechanical issues led to three instances of delayed reporting from the ambulance transfer surveillance system. There was at least a day’s delay in reporting from the official notifiable disease surveillance system because the public health centre was occupied with the COVID-19 outbreak response. From 23 July, these delayed cases were reflected in the daily report as soon as they were registered. Additionally, some delays in reporting occurred on weekends. This was due to processing delays at the office that receives reports on behalf of the public health centre during nights, weekends and holidays when the centre is closed. However, the delays did not affect the daily reporting procedures. Operation of the multisource surveillance system required 12 TMG staff members.

### Results of surveillance

#### Official notifiable diseases surveillance

**Fig. 3:**
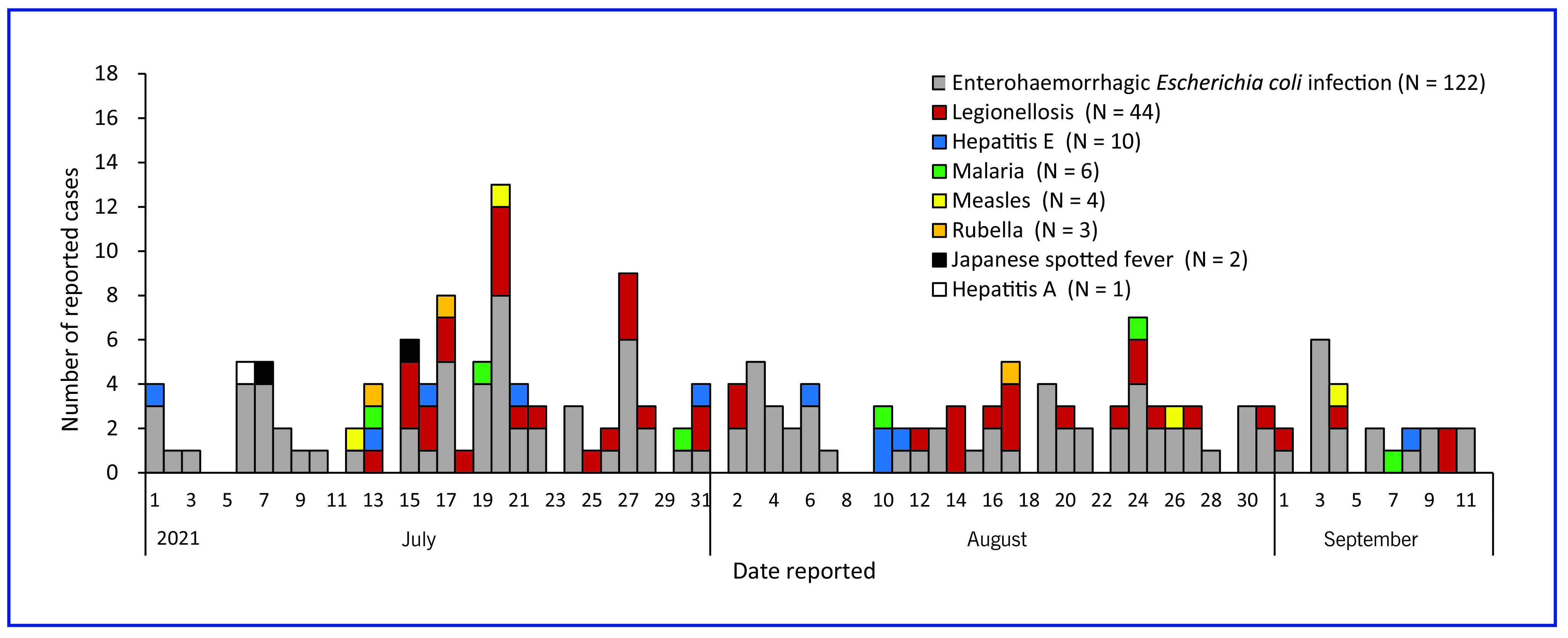
Daily reported cases from the notifiable diseases surveillance system during the Tokyo 2020 Olympic and Paralympic Games, 1 July–12 September 2021

There were 192 cases reported from medical facilities in Tokyo through the notifiable diseases surveillance system. This included 122 enterohaemorrhagic *Escherichia coli* infection cases and 44 legionellosis cases (**Fig. 3**). There were six malaria cases, including two cases related to the Games where the patients were infected abroad before arriving in Japan. Four suspected measles cases and three suspected rubella cases were reported, which subsequently tested negative. The other reports included 10 cases of hepatitis E, two of Japanese spotted fever and one of hepatitis A. The data reported through the notifiable diseases surveillance system did not indicate any unusual occurrence that could be related to the Games.

[**insert** Figure 3]

#### Official sentinel surveillance

**Fig. 4:**
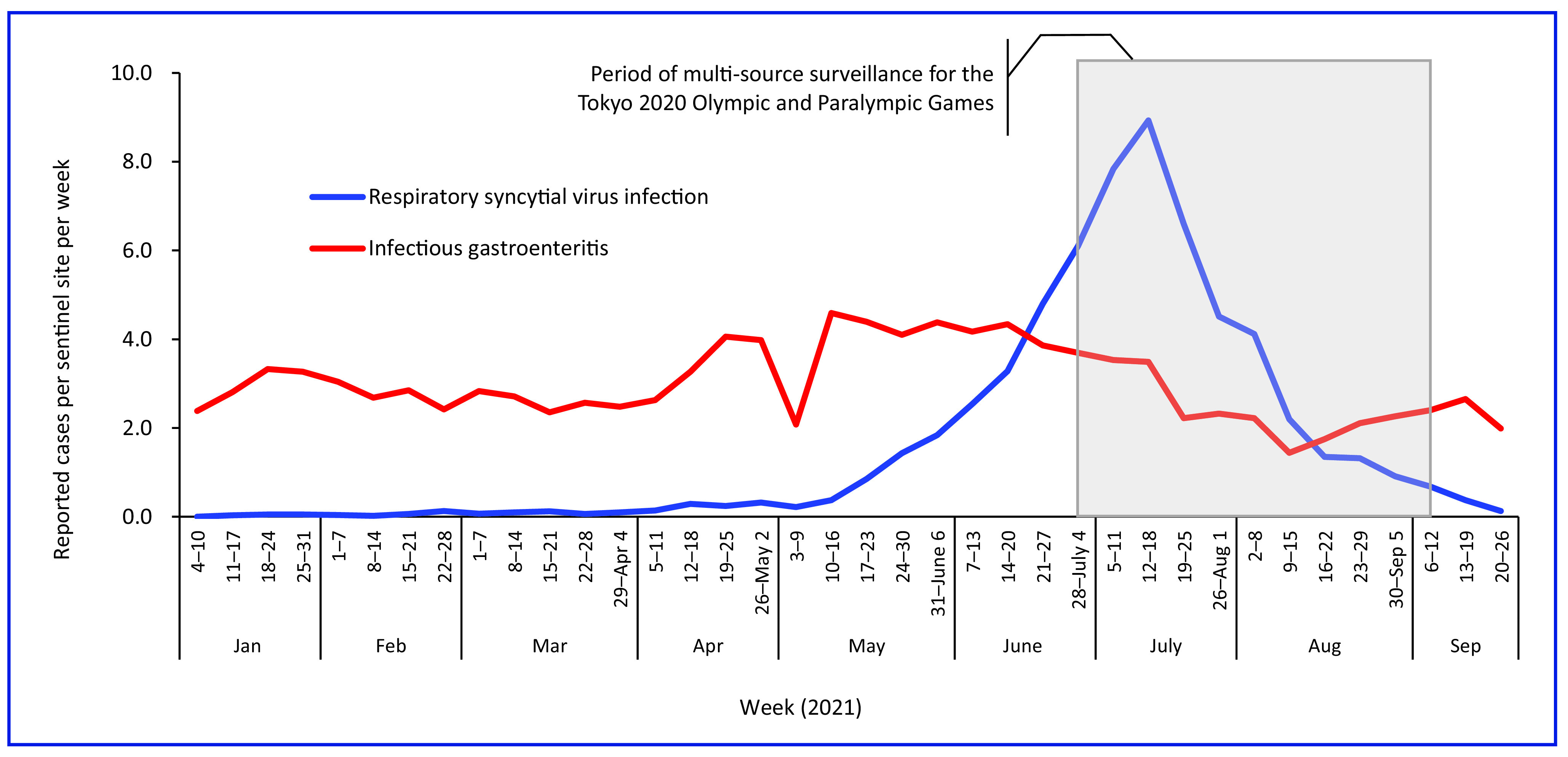
Number of cases per sentinel site per week for respiratory syncytial virus infection and infectious gastroenteritis from the sentinel surveillance system in Tokyo, 4–10 January to 20–26 September 2021

Respiratory syncytial virus (RSV) infection was the most frequently reported condition through the sentinel surveillance system during the Games, followed by infectious gastroenteritis (**Fig. 4**). A record high of 8.93 cases of RSV infection was reported per sentinel site in week 28 (12–18 July 2021). The number of reported cases per sentinel site per week of infectious gastroenteritis was 1.44–3.69 during the Games (**Fig. 4**).

[**insert** Figure 4]

#### Official syndromic surveillance

No report was made through the official syndromic surveillance system during the Games.

#### Cluster surveillance

There were 276 clusters of RSV infection and 29 clusters of infectious gastroenteritis reported through cluster surveillance during the Games. Most cases of these two infectious diseases were reported from nursery schools. There were also two clusters of adenovirus infection and two clusters of herpangina reported. Single clusters of *Clostridioides difficile* infection, haemolytic streptococcus infection, hand, foot and mouth disease, scabies and varicella were also reported. Each of the six clusters contained multiple infectious diseases. Seventy-seven clusters were undiagnosed from nursery schools and kindergartens. However, given the ongoing RSV epidemic and the reported symptoms of mainly fever, cough and nasal mucus, the public health centres attributed the cases to RSV infections.

**Fig. 5:**
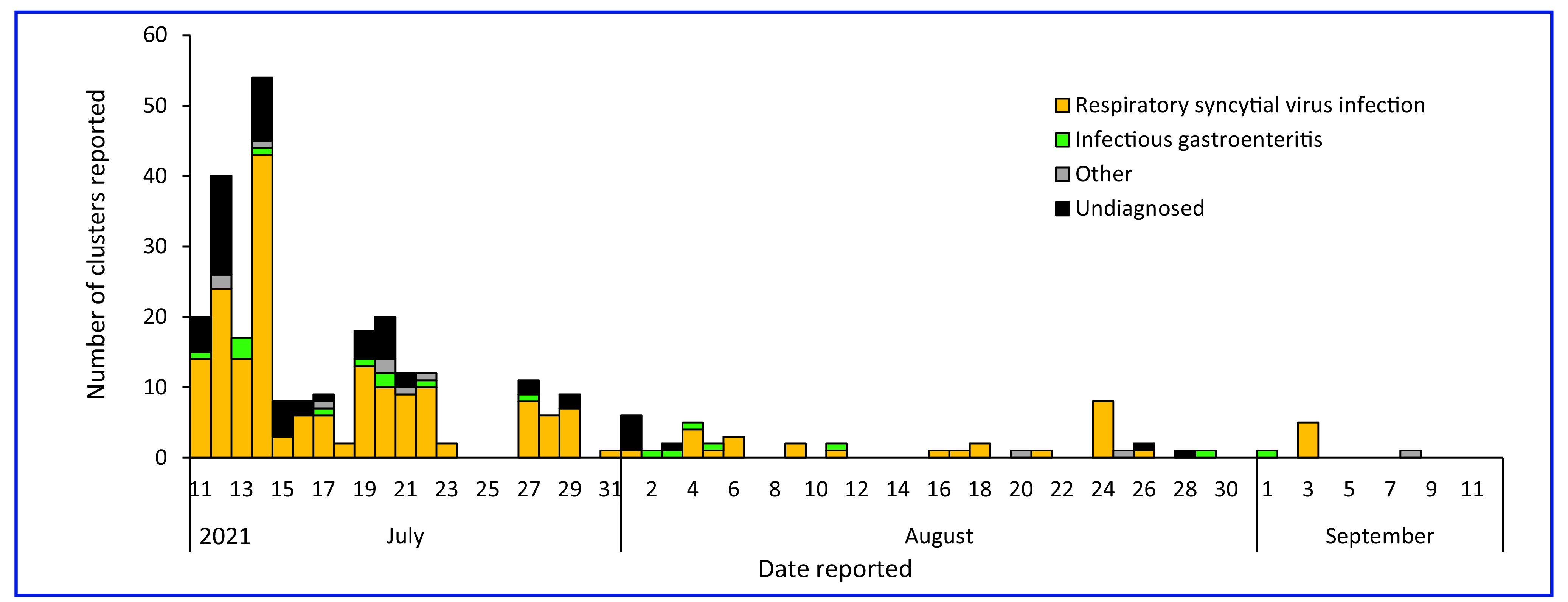
Number of clusters reported from the cluster surveillance system during the Tokyo 2020 Olympic and Paralympic Games, 11 July–12 September 2021

**Fig. 5** shows the daily number of clusters reported from 11 July to 12 September 2021. Due to daily reports in cluster surveillance being issued from 11 July, there were no daily data for 1–10 July. Nevertheless, clusters reported during 1–10 July included 67 clusters of RSV infection, 11 clusters of infectious gastroenteritis, four other clusters and 17 undiagnosed clusters.

[**insert** Figure 5]

#### Ambulance transfer surveillance

Five aberrant cases that required follow up were detected through the ambulance transfer surveillance and reported during daily reports on 6, 16, 20 July and 22 August 2021 (**Table 3**). Three cases were presumably attributable to infectious gastroenteritis among members of the same family while the other two were COVID-19 patients who were transferred from COVID-19 residential treatment facilities to hospital. No outbreak was observed.

**Table 3:** Aberrations detected in ambulance transfer surveillance during the Tokyo 2020 Olympic and Paralympic Games, by detection and reporting date, 6, 16, 20 July and 22 August 2021

Detection and reporting date	Situation	Investigation required at medical institution	Reason for not requiring investigation	Follow-up
6 July 2021	On 5 July at 14:00, three people in a city ward were rushed to the emergency department with fever, diarrhoea and vomiting	Not required, but monitoring was continued	All cases had mild symptoms and were presumed to be from one family	No further aberration
16 July 2021	On 15 July at 01:00, five people in a city were rushed to the emergency department with diarrhoea and vomiting	Not required, but monitoring was continued	All cases had mild symptoms and were presumed to be from one family	No further aberration
20 July 2021	On 19 July at 09:00, three people in a city were rushed to the hospital for fever and vomiting	Not required, but monitoring was continued	All cases were presumed to be from one family	No further aberration
22 August 2021	On 21 August at 10:00–11:00, three fever cases with COVID-19 were transported to medical facilities	Not required	The patients were being transported from a COVID-19 residential treatment facility to hospital	Not required
22 August 2021	On 21 August at 11:00–20:00, five fever cases with COVID-19 were transported to medical facilities	Not required	The patients were being transported from a COVID-19 residential treatment facility to hospital	Not required

#### Tokyo Infectious Alert system

No reports were made through the Tokyo Infectious Alert system during the Games.

#### Prescription surveillance

No aberration was reported through prescription surveillance during the Games.

#### Nursery School Absenteeism Surveillance System

No aberration requiring investigation was reported through (N)SASSy during the Games.

### Response

No investigations or public health responses were required during the Games.

## Discussion

The multisource surveillance conducted for the Games in Tokyo in 2021 confirmed that no cases of infectious disease were reported that required a specific public health response. Although there were diseases reported through the official notification and sentinel systems in Tokyo, none were assessed as being associated with the Games. Despite the Games being postponed to 2021 because of the COVID-19 pandemic, TMG was able to prepare the multisource surveillance system in just 3 months and successfully operate it during the Games, incorporating lessons learned from the 2013 Sports Festival in Tokyo and the 2019 Rugby World Cup.

The countermeasures implemented to prevent transmission during the COVID-19 pandemic contributed to the low activity of other infectious diseases (*11*, *12*) and might explain why no outbreak was detected during the Games. In addition, the risks posed by mass gatherings at the Games were reduced due to the fact that only players and staff members were in attendance.

No reports were received through the official syndromic surveillance system or the Tokyo Infectious Alert system during the Games. As null reporting was not required in the official syndromic surveillance system, it is not known whether the lack of reports was due to a true lack of illness or to issues with the system itself, such as unclear case definition or insufficient awareness within medical institutions to report. Further investigation into the reasons for non-reporting and the modifications to rectify them is necessary before the official syndromic surveillance system is used for a similar event.

The prescription surveillance or (N)SASSy coverage in Tokyo was not as complete for the Games as it had been historically. This surveillance system is part of routine automatic surveillance that is conducted irrespective of high-profile and mass-gathering events. Historical data can, therefore, be used for comparison during mass-gathering events to detect aberrations. The lack of aberrations from (N)SASSy was probably because the Games were held during the school summer holidays, when the risk of outbreaks was low. Although these systems did not report any aberrations during the Games, they are still expected to be helpful for future mass-gathering surveillance because they can provide valuable information collected with no effort or cost.

The cluster surveillance system comprised medical institutions and residential aged-care facilities that were not covered by other surveillance systems. Although the cluster surveillance worked well, the reported data were not linked to illness at the Games. This was because hospitalized patients and residents at care facilities were unlikely to come into contact with participants of the Games. Therefore, the monitoring of clusters in these populations might not be useful for future surveillance during the Games. The cluster surveillance system was incorporated into the multisource surveillance system for the Games due to the possibility that medical staff and professional caregivers may have worked as volunteers for the Games and could have transmitted infectious diseases, which was not the case. This occurred during the PyeongChang 2018 Winter Olympic Games, where 172 cases of norovirus were observed in volunteers who stayed at hostels, with four cases of diarrhoea in the Olympic villages. (*13*)

The daily reports of the surveillance data and risk assessments from the Games were not shared publicly by TMG. This contrasts with the enhanced syndromic surveillance for the National Sports Festival in Wakayama (*14*) and the Worldwide Uchinanchu (persons of Okinawan origin) Festival, (*15*) where all information and related risk assessments were posted online promptly. The Infectious Diseases Control Law requires that national and local governments analyse and disseminate information relating to infectious diseases to prevent transmission and outbreaks. However, TMG did not rapidly share the surveillance information with the general public during the Games as it did not have sufficient time for coordination with relevant organizations. This shortcoming should be improved to further multisource surveillance for mass gatherings such as the Games.

Communication between TMG and NIID was useful to multisource surveillance for the Games. The daily web conferences held with NIID facilitated information sharing, which contributed to risk assessment. The establishment of a system of collaboration with NIID during the planning and preparation stages for future mass-gathering or politically high-profile events in Tokyo is recommended. Both cooperation and communication among stakeholders including sections within TMG, event operators, the national government and other local governments involved in future events are also needed.

The multisource surveillance system used for the Games is likely to be used by TMG for future mass gatherings in Tokyo. As multisource surveillance increases the number of information sources being assessed, it may enable earlier detection and quicker response times than the routine surveillance conducted by TMG. It combines syndromic, cluster and laboratory information in a multilayered way to provide a comprehensive assessment of infectious diseases occurring during mass-gathering events. However, the content, surveillance systems used, reporting schedules and responses to the outbreak detected in the multisource surveillance must be tailored to each event.

There are some limitations to this analysis. It was limited to systems operated by TMG and did not include surveillance conducted by other local government jurisdictions where some of the events were held. Thus, any infectious diseases from these jurisdictions related to the Games would have been missed by the TMG system. Additionally, because of the short preparation time, we did not collaborate with related departments such as food and environmental divisions of TMG, the Tokyo Metropolitan Police Department and quarantine stations. If these agencies had detected public health events such as an outbreak of food poisoning or bioterrorism, then our multisource surveillance may not have detected it. Food poisoning information and mosquito surveillance are expected to be increasingly important infection control measures during multisource surveillance of other events held in the summer months. These are the challenges for future efforts in multisource surveillance by TMG.

In conclusion, the multisource surveillance undertaken during the Games by TMG resulted in no confirmed case of infectious disease requiring a response. This is likely due to there being no audience presence during the Games, and to the public health and social measures implemented for the COVID-19 pandemic. Our experience from earlier mass-gathering events contributed to our being able to establish multisource surveillance for the Games rapidly. Further lessons learned during the Games that can be applied to future mass-gathering events are to ensure the quality of the surveillance systems, include mechanisms for informing the general public in a timely manner, improve the sharing of surveillance data conducted by other local governments, and secure the necessary collaboration with related departments within TMG.
